# Artificial Intelligence in Periodontology: A Scoping Review

**DOI:** 10.3390/dj11020043

**Published:** 2023-02-08

**Authors:** James Scott, Alberto M. Biancardi, Oliver Jones, David Andrew

**Affiliations:** 1 School of Clinical Dentistry, The University of Sheffield, Claremont Crescent, Sheffield S10 2TA, UK; 2 Department of Infection, Immunity and Cardiovascular Disease, Polaris, 18 Claremont Crescent, Sheffield S10 2TA, UK

**Keywords:** periodontology, artificial intelligence, convolutional neural networks, radiography

## Abstract

Artificial intelligence (AI) is the development of computer systems whereby machines can mimic human actions. This is increasingly used as an assistive tool to help clinicians diagnose and treat diseases. Periodontitis is one of the most common diseases worldwide, causing the destruction and loss of the supporting tissues of the teeth. This study aims to assess current literature describing the effect AI has on the diagnosis and epidemiology of this disease. Extensive searches were performed in April 2022, including studies where AI was employed as the independent variable in the assessment, diagnosis, or treatment of patients with periodontitis. A total of 401 articles were identified for abstract screening after duplicates were removed. In total, 293 texts were excluded, leaving 108 for full-text assessment with 50 included for final synthesis. A broad selection of articles was included, with the majority using visual imaging as the input data field, where the mean number of utilised images was 1666 (median 499). There has been a marked increase in the number of studies published in this field over the last decade. However, reporting outcomes remains heterogeneous because of the variety of statistical tests available for analysis. Efforts should be made to standardise methodologies and reporting in order to ensure that meaningful comparisons can be drawn.

## 1. Introduction

Artificial intelligence (AI) aims to develop computer systems that can mimic human behaviour using machines. Within medicine and dentistry, commentators predicted as early as the 1970s that AI would bring clinical careers to an end [1]; however, this has not been the case. Science fiction will present AI as a comprehensive overarching intelligence [2], but this is far from the truth. Thus far, AI development has proved successful in solving problems in specific areas by learning distinct thinking mechanisms and perceptions.

Periodontitis is the sixth most prevalent disease worldwide. It is characterised by microbially associated, host-mediated inflammation that results in loss of alveolar bone and periodontal attachment, which can lead to tooth loss [3]. This disease has a well-reported but complex relationship with a number of other physiological systems leading to detrimental effects on quality of life and general health [4]. Further to this, a bi-directional relationship between systemic conditions, including chronic inflammatory disease such as diabetes [5,6] and atherosclerosis [7], has been shown. 

Periodontitis is also challenging for clinicians to accurately recognise and diagnose [8]. Best practice currently focuses on measuring soft tissues with a graduated probe [9] and assessing hard tissues with radiographic imaging [10]. However, these methods have poor inter- and intra-operator reliability due to variations in probing pressure and radiographic angulation [8].

As such, the study of periodontitis presents a diagnostic challenge linked to a disease process of complex relationships between predisposing factors that are difficult for clinicians and scientific processes to fully comprehend. These complex factors lend the study of this disease to the application of AI to best comprehend how these factors affect the diagnostics or understanding of its aetiology.

It is important to differentiate AI from traditional software development. In the traditional approach to software development, the researchers identify a series of processing steps and, optionally, a data-dependent strategy to reach the results. This is best described as an input ‘A’ is received, it is computed through the pre-defined strategy of sub-tasks, and an output ‘B’ is returned. As such, whilst this performs incredibly useful tasks for humankind, it requires vast amounts of effort to perform complex tasks and risks providing only limited availability for adaptation to unseen scenarios. Artificial intelligence, in contrast, has a different mode of work. When developing an AI-based tool, both the input ‘A’ and the required output ‘B’ are provided; the AI approach will then tune the tool to leverage the link between inputs and outputs, which can then be used on new (unseen) data sets, typically with remarkable performance [11]. 

AI is ever-increasing in medicine and dentistry as an assistive tool, becoming a central tenet in providing safe and effective healthcare. More recently, deep learning (DL) has been the mainstay of this endeavour, mainly through its applications stemming from the use of artificial neural networks (ANN) that exhibit a very high degree of complexity [12], where large numbers of artificial neurons (or nodes) are connected into layers and several hundreds, or thousands, of layers are assembled into specific structures called architectures. DL networks can assess large volumes of data to perform specific tasks, among which electronic health records, imaging data, wearable-device sensor collections, and DNA sequencing play a prominent role. Within medical fields, these are classically used for computer-aided diagnosis, personalised treatments, genomic analysis, treatment response assessment.

When images are used as input data, we as humans perceive digital images as analogues or as a continual flow of information. Still, a digital (planar) image is nothing more than a collection of millions of tiny points of colour or pixels, each with their 2D location. Thus, these pixel series can be viewed as strings of values with additional information about their neighbouring locations that software can process efficiently. 

A subset of ANNs, a convolutional neural network (CNN), is specifically designed for handling imaging data. The CNN concept was developed to replicate the visual cortex and differentiate patterns in an image [13]. Classic neural networks typically need to consider each pixel individually to process an image and therefore are heavily constrained in the size of the images that can be analysed. CNNs, on the other hand, are capable of working with the image data in their spatial layout; their output is a new set of data replicating the original layout of the image while increasing or condensing the information stored at each location. This process is similar to applying several digital filters to an image to ‘highlight’ key features that will collectively help perform the task at hand, e.g., select distinct aspects of an object to identify its presence inside the image. 

CNN-based architectures will often have multiple layers, or multiple levels at which these transformations are applied. Early layers will focus on picking up gross content such as edges, gradient orientation, and colour, with later layers focusing on higher-level (more task specific) features. This kind of approach is usually called an encoder because the iconic information inside the image is transformed into a more abstract, symbolic representation, and this is achieved by juxtaposing CNN-based blocks that progressively reduce the size of the image being processed while, concurrently, increasing the number of the channels, i.e., the number of values associated with each image pixel. The complementary approach to an encoder is a decoder where the abstract information is transformed into an iconic representation by successively increasing the image size while reducing the channel number. A common pattern in ANN architectures based on CNN layers is to have an encoder section, a decoder section, or both; for instance, the U-Net architecture [14], which is one of the most used approaches when segmenting imaging data, is structured as an encoder section followed by a decoder section to achieve a transformation of the image information from iconic to abstract and then back to iconic while performing the task at hand.

Therefore, CNNs can be taught to recognise specific collections of pixel/location pairs and subsequently find similar patterns in new image datasets. For CNNs to perform this function, they require a so called ‘training’ stage. Training is a process whereby humans identify target subsets and show a CNN what to look for. In image analysis, this often relies on experts labelling image sections of interest so that CNN can find similar regions in the future. For example, radiologists would draw around these nodules for a CNN to recognise nodules on CT images of a lung to show their correct extent. As the CNN is shown more nodules, it will become more capable of identifying similar regions. This process, leading to the software’s ability to carry out the nodule localisation independently, is called supervised learning [14].

Whilst in some cases CNNs can be definitive in their image recognition, they are more commonly used as assistive tools, whereby AI can highlight the areas that are likely to contain the sought pathology or image type. This has been demonstrated in radiology (for detecting abnormalities within chest X-rays) [15], dermatology (to detect lesions of oncological potential) [16], and ophthalmology (to detect specific types of retinopathy) [17]. It has been suggested, however, that within these fields, some of the work may lack the robustness to be truly generalisable to all clinical situations or, indeed, to be as accurate as medical professionals [18].

Within dentistry, radiographs are combined with a full clinical examination and special tests to aid in assessment, diagnosis, and treatment planning. The type of radiograph taken depends upon the disease or pathology being investigated or the procedure being undertaken, and may include bitewing, periapical, or orthopantomography [19]. Common justifications for taking dental radiographs include the diagnosis of dental caries staging and grading of periodontal disease [20], detection of apical pathology [21], or in the assessment of peri-implant health. A dentist will then report on these images. Still, it has been shown that in detecting both dental caries and periodontal bone loss, inter-ratee and intra-rater agreement is poor [22,23], lending this analysis to CNN assistance.

Image-based diagnostics is not the only area in which CNNs are currently used within medicine and dentistry. AI’s ability to assess large volumes of data for regression purposes lends itself to data analysis within larger data fields where traditional methods struggle. In medicine, success has been achieved using patient health records and metabolite data to predict Alzheimer’s disease, depression, sepsis, and dementia [11]. As the data processing technology has improved, specific to medicine and dentistry, a term introduced by the American Medical Association is that of ‘augmented intelligence’. This describes a conceptualisation of AI in healthcare, highlighting its assistive role to medical professionals.

Whilst the benefits of utilising AI within healthcare can be clear to see, for example reducing human error, assisting in diagnosis, and streamlining data analysis and task performance, which may ultimately lead to more efficient and cost-effective services, its adoption is not without issue. Specifically, these can include a lack of data curation, hardware, code sharing, and readability [24], as well as the inherent issues of introducing any new technology within a service such as reluctance to change, embedding new technology within current infrastructures, and ongoing cost maintenance. More recent criticism addressed the presence of implicit biases in the training datasets and the direct consequences in AI performance [25].

Within periodontology and implantology, AI is still in its relative infancy and has not yet been used to its full potential. With the advantages of diagnostic assistance, data analysis, and detailed regression, it would appear that much could be gained through applying this tool. Given the relative paucity of literature in the subject area, this scoping review aimed to assess the current evidence on the use of artificial intelligence within the field of periodontics and implant dentistry. This would both describe current practice and guide further research in this field.

## 2. Materials and Methods

This prospective scoping review was conducted by considering the original guidance of Arksey et al. [26] and more recent guidance from Munn et al. [27].

### 2.1. Focused Question and Study Eligibility 

The focused question used for the current literature search was “What are the current clinical applications of machine learning and/or artificial intelligence in the field of periodontology and implantology?”

The secondary questions were as follows:

1. Which methods were used in these studies to establish datasets; develop, train, and test the model; and report on its performance?
2. In cases where these models were tested against human performance:
  a. What metrics were used to compare performance?
  b. What were the outcomes?

The inclusion criteria for the studies:

1. Original articles published in English.
2. Implant- and periodontal-based literature using ML or AI models for diagnostic purposes, detection of abnormalities/pathologies, patient group analysis, or planning of surgical procedures.
3. Study designs whereby the use of ML or AI was used as the independent variable.

The exclusion criteria for the studies:

1. Studies not in English.
2. Studies using classic software rather than CNN derivative protocols for machine-based learning.
3. Studies using AI for purposes other than periodontology and peri-implant health.

### 2.2. Study Search Strategy and Process

**Figure 1:**
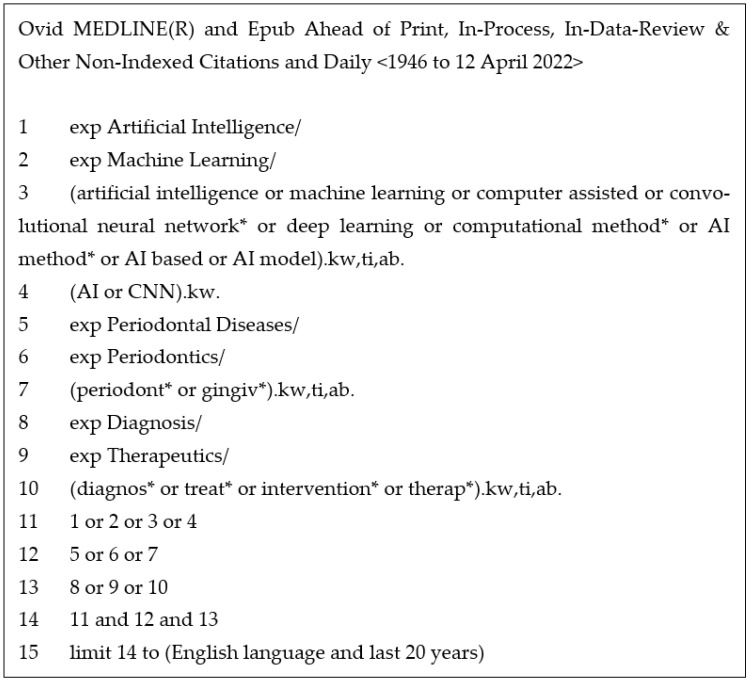
Medline via OVID search strategy.

An electronic search was performed via the following databases:

- Medline—the most widely used medical database for publishing journal articles. The search strategy for this is outlined in Figure 1.
- Scopus—the largest database of scientific journals.
- CINAHL—an index that focuses on allied health literature.
- IEEE Xplore—a digital library that includes journal articles, technical standards, conference proceedings, and related materials on computer science.
- arXiv—arXiv is an open-access repository of electronic preprints and postprints approved for posting after moderation but not peer review.
- Google Scholar—Google Scholar is a freely accessible web search engine that indexes the full text or metadata of scholarly literature across an array of publishing formats.

This electronic search was supplemented with hand searches of included texts references lists. The search strategy was compiled in collaboration with the librarian at the University of Sheffield Medical Library. Keywords were a combination of Medical Subject Headings (MeSH) terms and frank descriptors, which were employed to reflect the intricacies of each database. The publication period was set to 20 years and was restricted to literature in English. All articles were included for initial review in line with the scoping review methodology. Records were collated in reference manager software (Endnote^TM^; Version: 20, Clarivate Analytics, New York, NY, USA) [28], and the titles were screened for duplicates.

### 2.3. Study Selection

A single reviewer screened titles and abstracts. For records appearing to meet inclusion criteria, or where there was uncertainty, full texts were reviewed to determine their eligibility. This was completed twice by the reviewer at a 2-month interval to assess intra-rater agreement. Additional manual hand searching was performed of included full-text articles with reference lists from these studies included. These selected full texts were similarly read, and suitability for inclusion was as per the original criteria.

### 2.4. Data Extraction and Outcome of Interest

Data was extracted from the studies and recorded in a tabulated form. The standardised data collation sheet included the author title, year of publication, data format, application of ML/AI technique, the workflow of the ML/AI model, the subsequent training/testing datasets, the validation technique, the form of comparison used, and then some description of the performance of the AI model. The primary outcome of interest was the scope of current clinical applications of ML/AI in the field of periodontology and peri-implant health and the performance of these AI models in clinician or patient assistance. As this was a scoping review, all texts meeting eligibility criteria were subject to qualitative review.

## 3. Results

### 3.1. Study Selection and Data Compilation 

**Figure 2:**
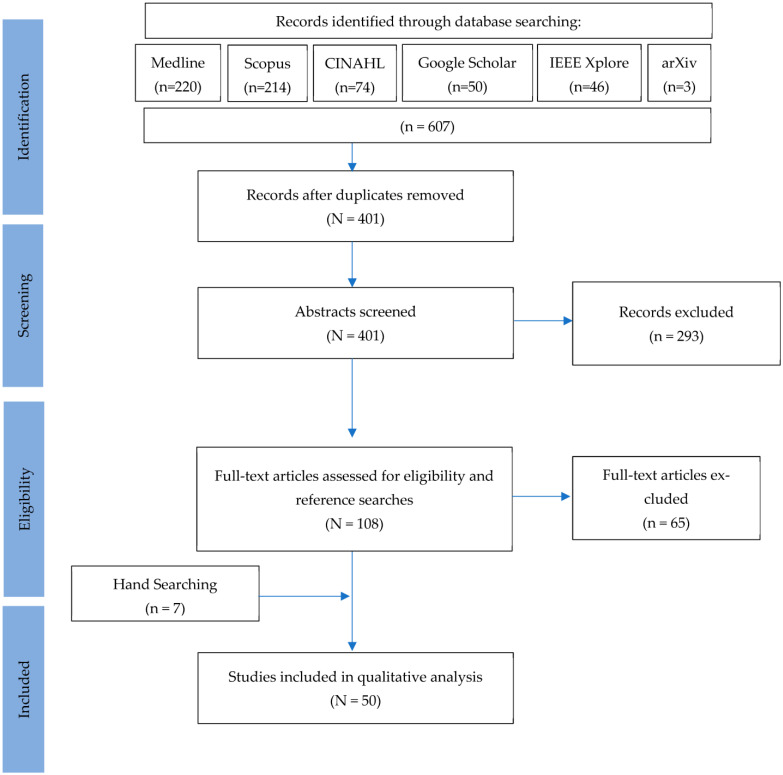
Study selection flowchart.

The study selection process is outlined in Figure 2. A total of 401 articles were identified for screening after the removal of duplicates. In total, 293 texts were excluded after screening for factors not meeting inclusion criteria. Full-text review and hand searching identified 50 studies for inclusion in the qualitative analysis. Most of these excluded articles tested software rather than an AI architecture to assess the input data. 

The included articles were appraised with key information compiled into a single data table (Table 1) for display in text format. 

### 3.2. Location of Research

**Figure 3:**
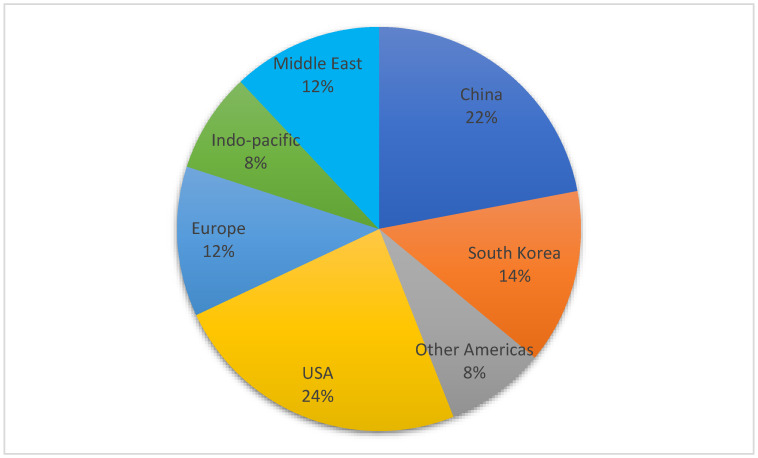
Location of research.

The first authors were from a wide variety of locations, illustrated in Figure 3. However, over half were from institutions in the USA (*n* = 12), China (*n* = 11), or South Korea (*n* = 7). The remainder were from Europe (*n* = 6), the Middle East (*n* = 4), the Indo-Pacific (*n* = 4), Brazil (*n* = 3), Turkey (*n* = 2), and Canada (*n* = 1). There is also strong evidence of international collaboration, with the first and last authors’ locations being geographically disparate in a number of cases (*n* = 8). 

### 3.3. Year of Publication 

**Figure 4:**
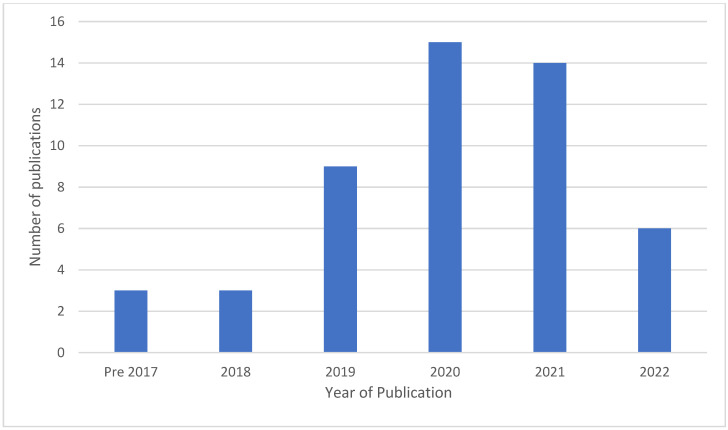
Publication of research.

Figure 4 depicts the year that studies were published. The first was in 2014, with a steady increase in the frequency of publication over the next decade. This number appears to be stabilising at 14–15 per year post-2020, in line with six found in 2022 prior to April when the searches were conducted.

### 3.4. Input Data 

**Figure 5:**
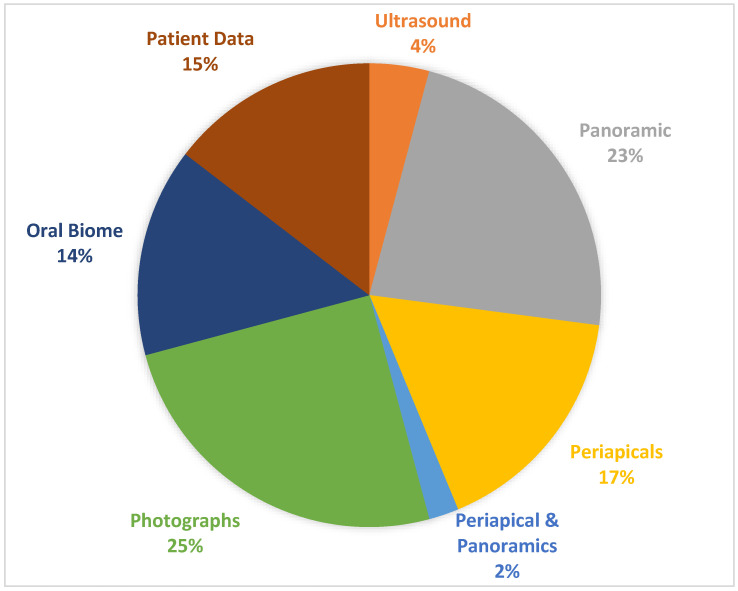
Input data.

All included studies had a periodontal focus; however, the input data varied significantly (Figure 5). The majority (68%) of studies focused on imaging data, using either photographs (*n* = 12), radiographs (*n* = 20), or ultrasound images (*n* = 2). Patient data (Electronic Health Record) were used in to attempt to predict periodontal or dental outcomes (*n* = 7). Metabolites and saliva markers were used to classify, diagnose, and predict periodontal and dental outcomes (*n* = 7).

### 3.5. Datasets 

The dataset size had a large span due to the variability of inputs and outcomes of the study types. Datasets for image processing studies ranged from 30 to 12,179, with a mean of 1774 for solely panoramic radiographs, 1064 for solely periapical radiographs, and 1431 for photographs. Due to the novel nature of ultrasound imaging, the two included studies contained less data (*n* = 35 and 627). Patient datasets varied between 216 and 41,543 with no meaningful descriptive statistics due to outcome and methodological heterogeneity. For all imaging studies, a mean of 1666 images were used. 

**Figure 6:**
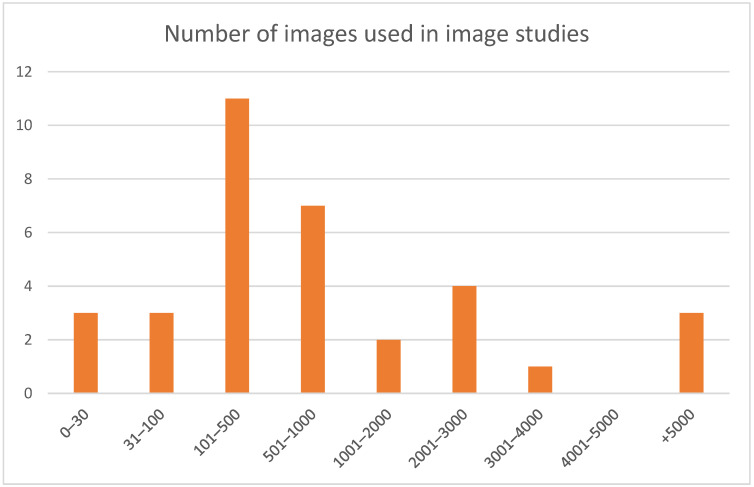
Numbers of images used in radiographic training datasets.

However, it is worth noting that the number of images included in studies with relatively heterogenous image types (i.e., plain film radiography) was not distributed in a Gaussian or normally distributed curve, with the median being significantly different to the mean. This was demonstrated with median (585 images) divergent from the mean (1940 images). Figure 6 shows the relative frequency peaks for visual analysis.

### 3.6. ML Architectures

A wide variety of convolutional architectures were used in this literature body (*n* = 67). The most common architecture described was a U-Net (*n* = 9). Several other architectures were used, including ResNet (*n* = 6), GoogLeNet Inception (*n* = 4), R-CNN or Faster R-CNN’s (*n* = 4), and AlexNet (*n* = 2). 

For image recognition, there appears to have been a shift to the use of U-Net over other architectures with all nine studies utilising or comparing this platform published in 2021 or 2022 [29,30,31,32,33,34,35,36,37]. When compared against other architectures, Dense U-Net out performed a standard U-Net [35], or U-Net was found to be optimised with a ResNet Encoder [36]. 

A number of studies involved patient data (e.g., metabolites or Electronic Health Record (EHR) data used support vector machine algorithms (SVM) [38,39,40,41,42,43]. The difference in data studies showed significant heterogeneity in methods, observations, and outcomes, and as such, no relevant statistical outcomes can be formed. However, descriptively, SVM showed either comparative outcomes [39] or reduced predictive capabilities [43] when compared to other ML formats such as ANN multilayer perceptron (MLP), random forest (RF), or naïve Bayes (NB).

### 3.7. Training and Annotation 

The majority of patient data research in this literature body focused on using CNNs to assist with regression analyses. In these cases, training is not required as the models are sourcing regression analyses rather than replicating human activity. 

In the case of image data processing, CNNs are mimicking humans and, as such, require training. General dentists performed training annotation in 47% of papers (*n* = 16), some form of specialist dentist or trainee specialist dentist performed training annotation in 16% of papers (*n* = 6), and radiologists in 8% (*n* = 3) of studies. Dental hygienists were also used (*n* = 1), as well as mixtures of clinicians (*n* = 6). 

The methods of labelling were, in the majority, manual annotation by drawing or labelling the external pixels of required features (*n* = 25). However, in a more recent paper [44], a process of ‘dye staining’ was used, whereby annotators merely highlighted areas of interest with a CNN used to ascertain characteristics around these single or multiple-point annotations had occurred. 

### 3.8. Outcome Metrics and Comparative Texts 

As is the nature of a scoping review there is vast heterogeneity in data forms, methodologies which result in very different outcome metrics [26,27]. These included an array of best fit measurements including F1 and F2 scores, precision, and accuracy, alongside sensitivity and specificity. Area under the curve analyses and ICC between test sets and representative expert labels were also frequently quoted. More specific imaging outcomes were also used with Jaccard’s Index, Pixel Accuracy, and Hausdorff Difference utilised. This made a meaningful statistical comparison of outcomes difficult due to the vast number of analyses presented.

Descriptively, as one would expect, when ML was asked to produce nominal outcomes, accuracy increased. In cases where outcomes were dichotomous, 90–98% accuracy was reported [45]. Within research that is more image focused, this is reflected when the task is more simple, such as in the work of Kong et al., with gross recognition of periodontal bone loss reporting an accuracy of 98% [46]. 

However, as the task asked of the ML increases in complexity, the accuracy was shown to drop. This is possibly best illustrated by the eloquent research of Lee et al. [36]. This study assessed several parameters to provide best outcomes for the radiographic staging of periodontitis, showing a U-Net with ResNet Encoder-50 for the majority of its image analysis. A power calculation was performed for gross bone loss. Here, Lee et al. describes greatest accuracy (0.98) where there was no bone loss, with reduction in accuracy (0.89) for fine increments such as for minimal bone loss (stage 1).

**Table 1:** Description of included studies.

	Study	Country	Year	Data Type	Subject Total	ML Architecture	Annotators	Performance Comparison	CNN Performance Comment	Brief Description
1	Papantonopoulos [45]	Greece	2014	Patient data	29	MLP ANN	n.a.	Not comparative	ANN’s gave 90–98% accuracy in classifying patients into AgP or CP.	ANNs used to classify periodontitis by immune response profile to aggressive periodontitis (AgP) or chronic periodontitis (CP) class.
2	Bezruk [47]	Ukraine	2017	Saliva	141	CNN (no description)	n.a.	Not comparative	Precision of CNN 0.8 in predicting gingivitis based upon crevicular fluid markers.	CNN was used for the learning task to build an information model of salivary lipid peroxidation and periodontal status and to evaluate the correlation between antioxidant levels in unstimulated saliva and inflammation in periodontal tissues.
3	Rana [48]	USA	2017	Photographs	405	CNN Autoencoder	Dentist	Not comparative	AU ROC curve of 0.746 for classifier to distinguish between inflamed and healthy gingiva.	Machine learning classifier used to provide pixel-wise inflammation segmentations from photographs of colour-augmented intraoral images.
4	Feres [41]	Brazil	2018	Plaque	435	SVM	n.a.	Not comparative	AUC > 0.95 for SVM to distinguish between disease and health. AUC for ability to distinguish between CP and AgP was 0.83.	SVM was used to assess whether 40 bacterial species could be used to classify patients into CP, AgP, or periodontal health.
5	Lee [49]	South Korea	2018	Periapical	1740	CNN encoder + 3 dense layers	Periodontist	Periodontist	CNN showed AU ROC curve of 73.4–82.6 (95% CI 60.9–91.1) in predicting hopeless teeth.	The accuracy of predicting extraction was evaluated and compared between the CNN and blinded board-certified periodontists using 64 premolars and 64 molars diagnosed as severe *n* the test dataset. For premolars, the deep CNN had an accuracy of 82.8%
6	Yoon [50]	USA	2018	Patient data	4623	Deep neural network-BigML	n.a.	Not comparative	DNN used as multi-regressional tool found correlation between ageing and mobility.	78 variables assessed by DNN were used to find a correlation that can predict tooth mobility.
7	Aberin [51]	Philippines	2019	Plaque	1000	AlexNet	Pathologists	Not comparative	Accuracy in predicting health or periodontitis from plaque slides reported at 75%.	CNN was used to classify which microscopic dental plaque images were associated with gingival health.
8	Askarian [52]	USA	2019	Photographs	30	SVM	n.a.	Not comparative	94.3% accuracy of SVM in detection of periodontal infection.	Smartphone-based standardised photograph detection using CNN to classify gingival disease presence.
9	Duong [31]	Canada	2019	Ultrasound	35	U-Net	n.a.	Orthodontist	CNN yielded 75% average dicemetric for ultrasound segmentation.	The proposed method was evaluated over 15 ultrasound images of teeth acquired from porcine specimens.
10	Hegde [39]	USA	2019	Patient data	41,543	SVM	n.a.	Not comparative	Comparison of ML vs. MLP vs. RF vs. SVM for data analysis. Similar accuracy found between all methods.	The objective was to develop a predictive model using medical-dental data from an integrated electronic health record (iEHR) to identify individuals with undiagnosed diabetes mellitus (DM) in dental settings.
11	Joo [53]	South Korea	2019	Photographs	451	CNN encoder + 1 dense layer	n.a.	Not comparative	Reported CNN accuracy of 70–81% for validation data.	Descriptive analysis of preliminary data for concepts of imaging analysis.
12	Kim [54]	South Korea	2019	Panoramic	12,179	DeNTNet	Hygienists	Hygienists	Superior F1 score (0.75 vs. 0.69), PPV (0.73 vs. 0.62), and AUC (0.95 vs. 0.85) for balanced setting DeNTNet vs. clinicians for assessing periodontal bone loss.	CNN used to develop an automated diagnostic support system assessing periodontal bone loss in panoramic dental radiographs.
13	Krois [55]	Germany	2019	Panoramic	85	CNN encoder + 3 dense layers	Dentist	Dentists	CNN performed less accurately than the original examiner segmentation and independent dentists’ observers.	CNNs used to detect periodontal bone loss (PBL) on panoramic dental radiographs.
14	Moriyama [56]	Japan	2019	Photographs	820	AlexNet	Dentist	Not comparative	Changes in ROC curves can have a significant effect on outcomes—looking at predicted pocket depth photographs and distorting images to improve accuracy.	CNN was used to establish if there is a correlation between pocket depth probing and images of the diseased area.
15	Yauney [57]	USA	2019	Patient data	1215	EED-net (custom net)	Dentist	Not comparative	AUC of 0.677 for prediction of periodontal disease based on intraoral fluorescent porphyrin biomarker imaging.	CNN was used to establish a link between intraoral fluorescent porphyrin biomarker imaging, clinical examinations, and systemic health conditions with periodontal disease.
16	Alalharith [58]	Saudi Arabia	2020	Photographs	134	Faster R-CNN	Dentist	Previously published outcomes	Faster R-CNN had tooth detection accuracy of 100% to determine region of interest and 77.12% accuracy to detect inflammation.	An evaluation of the effectiveness of deep learning based CNNs for the pre-emptive detection and diagnosis of periodontal disease and gingivitis by using intraoral images.
17	Bayrakdar [59]	Turkey	2020	Panoramic	2276	GoogLeNet Inception v3	Radiologist and periodontist	Radiologist and periodontist	CNN showed 0.9 accuracy to detect alveolar bone loss.	CNN used to detect alveolar bone loss from dental panoramic radiographic images.
18	Chang [60]	South Korea	2020	Panoramic	340	ResNet	Radiologist	Radiologists	0.8–0.9 agreement between radiologists and CNN performance.	Automatic method for staging periodontitis on dental panoramic radiographs using the deep learning hybrid method.
19	Chen [61]	China (and the UK)	2020	Photographs	180	ANN (no description)	n.a.	Not comparative	ANN accuracy of 71–75.44% for presence of gingivitis from photographs.	Visual recognition of gingivitis testing a novel ANN for binary classification exercise—gingivitis or healthy.
20	Farhadian [38]	Iran	2020	Patient data	320	SVM	n.a.	Not comparative	The SVM model gave an 88.4% accuracy to diagnose periodontal disease.	The study aimed to design a support vector machine (SVM)-based decision-making support system to diagnose various periodontal diseases.
21	Huang [40]	China	2020	Gingival crevicular fluid	25	SVM	n.a.	n.a.	Classification models achieved greater than or equal to 91% in classifying SP patients, with LDA being the highest at 97.5% accuracy.	This study highlights the potential of antibody arrays to diagnose severe periodontal disease by testing five models (SVM, RF, kNN, LDA, CART).
22	Kim [42]	South Korea	2020	Saliva	692	SVM	n.a.	n.a.	Accuracy ranged from 0.78 to 0.93 comparing neural network, random forest, and support vector machines with linear kernel, and regularised logistic regression in the R caret package.	CNN was used to assess whether biomarkers can differentiate between healthy controls and those with differing severities of periodontitis.
23	Kong [46]	China	2020	Panoramic	2602	EED-net (custom net)	Expert?	Expert?	The custom CNN performed better than U-Net or FCN-8, all with accuracies above 98% for anatomical segmentation.	CNN was used to complete maxillofacial segmentation of images, including periodontal bone loss recognition.
24	Lee [62]	South Korea	2020	Periapicals and panoramic	10,770	GoogLeNet Inception v3	Periodontist	Periodontist	The CNN (0.95) performed better than human (0.90) for OPGs, but the same for PAs (0.97).	CNN used for identification of implants systems and their associated health.
25	Li [63]	Saudi Arabia	2020	Panoramic	302	R-CNN	Dentist	Other CNNs and dentist	Proposed architecture gave accuracies of 93% for detecting no periodontitis, 89% for mild, 95% for moderate, and 99% for severe.	This study compared different CNN models for bone loss recognition.
26	Moran [64]	Brazil	2020	Periapicals	467	ResNet, Inception	Radiologist and dentist	Compares two CNN approaches for accuracy	AUC ROC curve for ResNet and Inception was 0.86 for identification of regions of periodontal bone destruction.	Assessment of whether a CNN can recognise of periodontal bone loss improve post-image enhancement?
27	Romm [65]	USA	2020	Metabolites	N/A	CNN (No description), PCA	n.a.	n.a.	Oral cancer identified rather than a periodontal disease with 81.28% accuracy.	CNN to analyse metabolite sets for different oral diseases to distinguish between different forms of oral disease.
28	Shimpi [43]	USA	2020	Patient data	N/A	SVM, ANN	n.a.	n.a.	ANN presented more reliable outcomes than NB, LR, and SVM.	The study reviewed classic and CNN regression to assess accuracy in prediction for periodontal risk assessment based on EHIR.
29	Thanathornwong [66]	Thailand	2020	Panoramic	100	Faster R-CNN	Periodontist	Periodontist	0.8 precision for identifying periodontally compromised teeth using radiographs.	CNN used to assess periodontally compromised teeth on OPG.
30	You [67]	China	2020	Photographs	886	DeepLabv3+	Orthodontist	Orthodontist	No statistically significant difference in the ability to discern plaque on photographs compared to clinician.	CNN used to assess plaque presence in paediatric teeth.
31	Cetiner [68]	Turkey	2021	Patient data	216	MLP ANN	n.a.	n.a.	The DT was most accurate, with accuracy of 0.871 compared to LR (0.832) and LP (0.852).	Assessment of three models of data mining to provide a predictive decision model for peri-implant health.
32	Chen [69]	China	2021	Periapicals	2900	r-CNN	Dentist	n.a.		CNN used to locate periodontitis, caries, and PA pathology on PAs.
33	Danks [70]	UK	2021	Periapicals	340	ResNet	Dentist	Dentist	Predicting periodontitis stage accuracy of 68.3%.	CNN used to find bone loss landmarks using different tools to provide staging of disease.
34	Kabir [30]	USA	2021	Periapicals	700	Custom CNN combining Res-Net and U-Net	Periodontitis	Periodontitis	Agreement between professors and HYNETS of 0.69.	CNN calibrated with bone loss on PAs applied to OPGs for staging and grading of whole-mouth periodontal status.
35	Khaleel [71]	Iraq	2021	Photographs	120	BAT algorithm, PCA, SOM	Dentist	n.a.	BAT method provided 95% accuracy against ground truth	Assessment of different algorithms’ efficacy in recognising gingival disease.
36	Kouznetsova [72]	USA	2021	Salivary metabolites	N/A	DNN	n.a.	n.a.	Model performance assessment only of different CNNs.	CNN predicts which molecules should be assessed for metabolic diagnosis of periodontitis or oral cancers.
37	Lee [35]	South Korea	2021	Panoramic	530	U-Net, Dense U-Net, ResNet, SegNet	Radiologists	Radiologists	The accuracy of the resulting model was 79.54%.	Assessment of a variety of CNN architectures for detecting and quantifying the missing teeth, bone loss, and staging on panoramic radiographs.
38	Li [73]	China	2021	Photographs	3932	Fnet, Lnet, cnet	Dentist	Dentists	Low agreement between three dentists and CNN in heatmap analysis.	CNN used for gingivitis detection photographs.
39	Li [29]	China	2021	Photographs	110	DeepLabv3+	Dentist	n.a.	MobileNetV2 performed in a similar manner to Xception65; however, Mob, was 20× quicker.	Different CNNs trialled for RGB assessment of gingival tissues to assess inflamed gum detection on photographs.
40	Ma [74]	Taiwan	2021	Panoramic	432	ConvNet, U-Net	Unknown	n.a.	ConvNet analysis post U-Net segmentation—the model showed moderate levels of agreement (F2 score of between 0.523 and 0.903) and the ability to predict periodontitis and ASCVD.	CNNs used to assess for atherosclerotic cardiovascular disease and periodontitis on OPGs.
41	Moran [75]	Brazil	2021	Periapicals	5	Inception and for super-resolution SRCNN	Dentist	n.a.	Minimal enhancement of CNN performance was noted from super resolution, which may introduce additional artefacts.	The study compared the effects of super resolution methods on the ability of CNNs to perform segmentation and bone loss identification.
42	Ning [76]	Germany	2021	Saliva	N/A	DisGeNet, HisgAtlas	n.a.	n.a.	DL-based model able to predict immunosuppression genes in periodontitis with an accuracy of 92.78%.	CNN to identify immune subtypes of periodontitis and pivotal immunosuppression genes that discriminated periodontitis from the healthy.
43	Shang [32]	China	2021	Photographs	7220	U-Net	Dentist	Dentist	U-Net to have a 10% increased recognition of calculus, wear facets, gingivitis, and decay	Comparison of U-Net vs. comparison between U-Net and DeepLabV3/PSPNet architecture for image recognition on oral pictures for wear, decay, calculus, and gingivitis.
44	Wang [77]	USA	2021	Metabolites	N/A	FARDEEP	n.a.	n.a.	ML successfully used in logistic regression of plaque samples.	CNN is used as a processing tool for clinical, immune, and microbial profiling of peri-implantitis patients against health.
45	Jiang [37]	China	2022	Panoramic	640	U-Net, YOLO-v4	Periodontist	Periodontist	Compared to the ground truth, accuracy of 0.77 was achieved by the proposed architecture.	CNN used to provide % bone loss and resorption/furcation lesion and staging of periodontal disease from OPGs.
46	Lee [36]	USA	2022	Periapicals	693	U-Net, ResNet	Dentist	Dentist	The accuracy of the diagnosis based upon staging and grading was 0.85	Full mouth PA films were used to review bone loss—staging and grading were then performed.
47	Li [73]	China	2022	Photographs	2884	OCNet, Anet	Dentist	Dentist	CNN provided AUC prediction of 87.11% for gingivitis and 80.11% for calculus.	Research trialling different methods of segmentation to assess plaque on photographs of tooth surfaces (inc ‘dye labelling’).
48	Liu [78]	China	2022	Periapicals	1670	Faster R-CNN	Dentist	Dentist	The results confirm the advantage of utilising multiple CNN architectures for joint optimisation to increase UTC ROC boosts of up to 8%.	CNN used to assess implant marginal bone loss with dichotomous outcomes.
49	Pan [33]	USA	2022	Ultrasound	627	U-Net	Dentist	Dentist	Showed a significant difference between CNN outcome and dental experts’ labelling.	CNN was used to provide an estimation of gingival height in porcine models.
50	Zadrozny [34]	Poland	2022	Panoramic	30	U-Net	Radiologists	Dentists	Tested CNN showed unacceptable reliability for assessment of caries (ICC = 0.681) and periapical lesions (ICC = 0.619), but acceptable for fillings (ICC = 0.920), endodontically treated teeth (ICC = 0.948), and periodontal bone loss (ICC = 0.764).	Testing of commercially available product Diagnocat in the evaluation of panoramic radiographs.

## 4. Discussion

CNNs are becoming increasingly clinically relevant in their ability to assess imaging data and can be an excellent utility for analysing large clinically relevant datasets. In the present review, we systematically compiled the application of CNNs in the field of periodontology, evaluating the application and outcomes of these studies. The majority of these studies were completed in the USA and China, which is line with the majority of DL papers in the medical spheres [79].

The use of CNNs in periodontal and dental research has continuously grown over the last decade. Since the first publications using CNNs in the early 2010s, there has been an exponential increase in the number of publications using this tool. In 2021 alone, there were 2911 registered studies on PubMed with CNN in the title, up from 42 in 2010. This, of course, makes empirical sense. The power, utility and applicability of this tool are endless and are improving as the architectures evolve, providing both generic and task-focused utility. 

Previous systematic and scoping reviews in dentistry have highlighted the underuse of this tool and a lag for dental research in this area [80,81]. However, with the total number of periodontal imaging papers alone now equalling the number of dental imaging papers in 2018, this lag is likely to have been overcome. This is unequivocally to the betterment of dental patient care when considering the benefits patients have enjoyed through similar endeavours in medicine [82].

The advantage of a broad search is the volume of literature that is assessed. However, it must be noted that a significant portion of the literature was derived from technical standards, conference proceedings, and related materials on computer-science-oriented repositories rather than journal articles. This is advantageous for the authors because the time to publication can be significantly reduced by removing the requirement for peer or public review. This may suit the rapidly evolving world of computer science, where breakthroughs can occur at breakneck speed, but it is unclear if the intrinsic validity of these publications is reduced due to a lack of public/peer scrutiny. This literature is published by technical scientists and therefore reported differently to how clinicians might expect it.

The majority of studies focused on the processing of images and the recognition of structures. Radiography accounted for two-thirds of these data (Figure 5), reasonably evenly split between periapical and panoramic radiographs. With both imaging modalities indicated in the assessment of patients with periodontitis, virtual assistance in diagnosis will be relevant to the clinician. In these studies, the majority of studies focused on image segmentation rather than pathology detection. We can only assume that this was due to the relative complexity of a detection tool compared to segmentation. However, moving forwards, relative detection from consecutive radiographs or more pathology identification would be of use as an assistive tool.

Database collation is an issue for all data science. This is of significant difficulty when compiling data from medical records. When considering the field of machine learning, suitable numbers of records are required to train and refine an AI tool. Supervised training is recognised as a central tenant to improve a CNN’s performance. This process requires that the CNN is shown labelled images to define structures for the CNN to segment. It is, however, possible to over-train CNNs, resulting in errors due to over-recognition. 

With homogenous data, it would not be unreasonable to believe that the distribution of training input utilised would be Gaussian or normally distributed. However, we find that the mean number of plain film radiographs utilised for training was multinomial in distribution. This is best illustrated by the divergent mean (1940) and median (585). Figure 6 shows a histogram of the data, where peaks can be seen between 100 and 1000 images and over 4000 images used. The authors query whether this was related to convenient sampling implicit with smaller numbers of data or whether this is reflective of the inherent variabilities of the requirements of the differing architectures of ML used. A power calculation was performed in a single paper, but only for a single outcome, and as such may not have offered the researcher team an accurate assessment of data volume required [37]. Further to this, there was no notable descriptive correlation between outcome-reported accuracy and the number of images used, with larger studies reporting a variety of accuracies or other outcomes [30,54,59,62,78]. This may be solely reflective of the noted variety of reporting outcomes and methodologies rather than due to a lack of correlation.

Labelling these images for training and subsequent reference tests is also of paramount importance. Gold standards were applied in several studies whereby more than one clinician or a specialist radiologist was used to perform this manual task to ensure a consensus approach to best fit was taken. However, in many studies, this was performed by a single evaluator, reducing both the external validity of the results due to single operator bias and the internal validity, introducing potential systematic error. Whilst some efforts have been made to standardise methodologies [83,84], these are still yet to be adopted or referenced in wider practice. The majority of studies used pixel-by-pixel annotation tools, with single study moving to ‘Dye Staining or ‘Grab Cut’ methodology [73]. This practice changes the digital annotation sequencing, essentially highlighting areas of interest to the CNN rather than circumscribing the anatomical feature of relevance. This is already mainstream in several other fields [85], possibly highlighting the digital technical lag present in dental research and the opportunities available in this field.

The performance of CNNs was reported in very heterogeneous manners, almost all of which come with drawbacks. Area under the curve (AUC) analyses are important, but only partially informative when it comes to outcomes. Lying above the curve is just a minimum requirement and, typically, only very elevated values are representative of applications suitable to handle real-world data; thus, sensitivity and specificity need to be reported as well to indicate performance. This was the case in some later papers, but those additional values were missing from most papers where AUC was a reported outcome. Accuracy was the most commonly reported outcome known to distort results when class imbalances are in place [86]. This is due to the class distribution being unknown in the training data, meaning that there is an assumption as to which population is more present (i.e., bone loss or no bone loss). This assumption skews data, making the reported ≈70–90% accuracy less meaningful due to inherent guesswork in ascertaining the class.

It has been suggested that the gold standard employed in these papers should be for models to be tested against independent expert assessors on truly unseen data, or indeed for the models to be used in a clinical trial [83]. However, no studies included compare the outcomes from a truly independent examiner team on unseen data against the proposed AI/ML outcomes. This leads to a ‘fuzzy gold standard’, whereby the AI/ML outcome is being marked against the clinician examiners that were used to train the tool. Until literature providing baseline information on the efficacy of examiners is universally accepted or studies showing performance against truly independent dentist performance in a clinical environment is shown, the assistive nature of these tools in a ‘real world setting’ will remain unproven.

With the marked reporting heterogeneity and uncertainty of gold standard testing, it is not surprising that gauging meaningful comparison and pooling data for meta-analysis has not been done. This may be indicated in a further systematic review in the future as more standardisation of this form of research occurs. However, descriptively, when considering outcomes, this body of research could show little improvement of accuracies over the last nine years, with accuracies of 90–98% reported in 2014 [45] and accuracies of 0.85 described in 2022. However, we feel that whilst the reported figures have remained similar, the question has markedly changed over this time. Ever increasingly complex problems are being asked of ML. Whilst historical papers asked more nominal questions, more recent literature such as Jiang et al. [37] looked to radiographically stage periodontitis from OPG radiographs. This represents a continuous problem that is grouped into ordinal datasets. Here, the accuracy established was similar to the periodontists who had completed the original manual labelling in the region of 0.85, depending upon tooth position and severity of bone loss. This shows a significant improvement in the quality of the outcome and indicates that as the power of ML increases, the assistive nature of these tools may become more powerful. 

This scoping review comes with its inherent limitations. The chosen question is as broad; as is inherent within the purpose of a scoping review [27], reflected by a broad search strategy. Whilst including articles from resources such as ArXiv offers the readers a larger pool of references, it should be noted that these articles are not peer-reviewed and therefore may lack some of the methodological rigour of published literature. In addition to this, the broad inclusion criterion has led to the authors somewhat controversially opting to include papers using a broad variety of machine learning and artificial intelligence modalities employed to analyse a broad range of data types. The resulting heterogeneity of the literature reduced the opportunity for meaningful outcome data comparison. However, the authors would add that they agree with the findings of referenced homogenisation efforts to reduce the variability in results expressed in this field. 

Methodologically, the main frailty revolves around searches and synthesis being performed by a single reviewer, with cursory checks by second and third authors. Whilst the single reviewer completed the synthesis twice with an extended time interval between reviews, this still forms an area of inherent bias. However, this was necessary with the practicality of this study.

## 5. Conclusions

Overall, this review gives insight into the application of machine learning in the field of Periodontology. Given artificial intelligence’s relative infancy in healthcare, it is not surprising that significant heterogeneity was found in the methodology and reporting outcomes. All efforts should be made to bring further research in line with increasingly recognised gold standard for research and reporting. International agreement on a gold standard against which to measure these tools would also significantly assist readers in assessing the utility of this modality of tool. As such, at this juncture, no accurate conclusions can be drawn as to the efficacy and usefulness of this tool in the field of periodontology.

## Data Availability

Further data required may be requested from the corresponding author.
